# Draft genome sequence of a novel species of *Radiobacillus* (strain PE A8.2) isolated from a red Antarctic seaweed

**DOI:** 10.1128/mra.01276-24

**Published:** 2025-03-06

**Authors:** Matías Goddard, Sergio Leiva

**Affiliations:** 1 Institute of Biochemistry and Microbiology, Universidad Austral de Chile, Valdivia, Chile; Montana State University, Bozeman, Montana, USA

**Keywords:** Antarctica, seaweed, epiphytic bacteria, *Radiobacillus*

## Abstract

We report the draft genome sequence of *Radiobacillus* sp. nov. PE A8.2, an endospore-forming bacterium isolated from the surface of the red seaweed *Pyropia endiviifolia* collected in King George Island, Antarctica. The genome assembly comprised 5,183,530 bp, with a 37.5% G+C content and 5,077 coding sequences.

## ANNOUNCEMENT

At present, little information is available on the epiphytic bacterial composition of Antarctic seaweed species. A few studies have reported that the surface of Antarctic seaweeds is colonized by a diverse Gram-positive bacterial microbiota, mostly Actinobacteria (1, 2). Reports on Firmicutes found on Antarctic seaweeds are scarce (3, 4). During a study aimed at investigating the diversity of culturable bacteria associated with Antarctic seaweeds, we isolated a Gram-positive, endospore-forming bacterium, designated as strain PE A8.2, from a specimen of the red seaweed *Pyropia endiviifolia* collected from the upper eulittoral zone of Rodriguez Point (62°11′57″ S, 58°56′34″ W), King George Island (South Shetland Islands, Antarctica Peninsula). The isolate was obtained from the surface of healthy fronds of *P. endiviifolia*. Initial 16S rRNA amplicon sequencing using the 27F and 1492R primers indicated that strain PE A8.2 belongs to the genus *Radiobacillus*. The genus *Radiobacillus* (*Firmicutes*, *Bacillaceae*) contains only two species, *Radiobacillus deserti*, an ultraviolet-resistant bacterium isolated from desert soil (5), and *Radiobacillus kanasensis*, a halotolerant bacterium isolated from woodland soil (6).

Strain PE A8.2 was isolated from the surface of *P. endiviifolia* on marine agar 2216 (BD) at 20°C. Growing colonies were selected and purified by streaking on 2216 marine agar three times. Culture purity was confirmed by Gram stain and consistency of colony characteristics. Genomic DNA was extracted from 72-h cultures grown in marine broth 2216 (BD) at 20°C using the GeneJET Genomic DNA Purification Kit (Thermo Scientific) and sequenced at AUSTRAL-omics, Valdivia, Chile.

A DNA library with an insert size of 420 bp was prepared using 90 ng of DNA and the MGIEasy FS DNA Library Prep Set (MGI Tech, China) according to the manufacturer’s instructions. The quality of the library was verified using the Agilent fragment analyzer and the DNF-474 kit. The Qubit DNA high-sensitivity assay kit in a Qubit Fluorometer (Thermo Fisher Scientific) was used for library quantification. Circularized DNA was prepared using the MGIEasy Circularization Kit (MGI Tech). DNA nanoballs (DNBs) were created using a DNBSEQ-G400RS High-throughput Sequencing Kit (MGI Tech). Whole-genome sequencing was performed on a DNBSEQ-G400 system (MGI Tech) using the PE150 (paired-end 2 × 150 bp) run format and produced a total of 3,285,052 reads. For all analyses, default parameters were used for all softwares unless otherwise specified. Raw reads quality was assessed with FastQC v0.11.5, and low-quality regions were removed using Trimmomatic v.0.39 (7). A *de novo* assembly was generated using Unicycler v0.4.8 (8) excluding contigs smaller than 1,000 bp. Genome annotation was performed with Rapid Annotations using Subsystems Technology server (9), assembly metrics were calculated with QUAST v5.0.2 (10), and completeness was assessed with BUSCO v1 (11) on the gVolante web server (12). Whole-genome average nucleotide identity based on BLAST (ANIb) and digital DNA-DNA hybridization (dDDH) values was calculated using the JSpeciesWS (13) and the Genome-to-Genome Distance Calculator (14), respectively.

The complete 16S rRNA gene was retrieved from whole genome data. EzBioCloud 16S database v.2023.08.23 (https://www.ezbiocloud.net/) (15) was used for sequence comparison. Strain PE A8.2 was found to exhibit 97% sequence similarity with *R. kanasensis* 80^T^ (GenBank accession number MT509021) and 96.5% with *R. deserti* TKL69^T^ (GenBank accession number MK760066), which indicates that this strain represents a novel species. The average nucleotide identity values between the PE A8.2 strain and *R. kanasensis* and *R. deserti* are 70.83% and 70.66%, respectively, and dDDH values between the PE A8.2 strain and *R. kanasensis* and *R. deserti* are 21.6% and 21%, respectively.

PE A8.2 reads were assembled in 110 contigs, with an average coverage of 167-fold and a N50 value of 84,270 bp. The genome size is 5,183,530 bp, with a 37.5% G+C content. The draft genome contains 5,077 coding sequences and 84 RNA genes. Assembly achieved a completeness of 95% (38/40 ortholog genes). Two plasmid partitioning genes, *parA* and *parB*, were detected, which suggests the occurrence of a plasmid.

## Data Availability

All data are available under BioProject accession number PRJNA1185888. The raw reads were deposited at the SRA under accession number SRS23219602. The GenBank accession number is JBJGXI000000000.The version described here is the first version.
